# Increased Concentration of Anti-Egg Albumin Antibodies in Cerebrospinal Fluid and Serum of Patients with Alzheimer’s Disease—Discussion on Human Serpins’ Similarity and Probable Involvement in the Disease Mechanism

**DOI:** 10.3390/biom15081085

**Published:** 2025-07-27

**Authors:** Dionysia Amanatidou, Magdalini Tsolaki, Vasileios Fouskas, Ioannis Gavriilidis, Maria Myriouni, Anna Anastasiou, Efthimia Papageorgiou, Diona Porfyriadou, Zoi Parcharidi, Eleftheria Papasavva, Maria Fili, Phaedra Eleftheriou

**Affiliations:** 1 School of Health, Department of Biomedical Sciences, International Hellenic University, Sindos, 57400 Thessaloniki, Greece; damanatidou@ihu.gr (D.A.); fvasillis79@gmail.com (V.F.); giannisgavriilidis2@gmail.com (I.G.); mariamyriouni@gmail.com (M.M.); zwhpar@gmail.com (Z.P.); epapasavva2000@gmail.com (E.P.); fili.maria@outlook.com (M.F.); 2 Laboratory of Neurodegenerative Diseases, Center for Interdisciplinary Research and Innovation (CIRI-AUTh), Balkan Center, Aristotle University of Thessaloniki, Building A, 57001 Thessaloniki, Greece; tsolakim1@gmail.com (M.T.); anna.ch.anastasiou@gmail.com (A.A.); e.pap97@gmail.com (E.P.); 3 Alzheimer Hellas, 13 Petrou Sindika st., 54643 Thessaloniki, Greece

**Keywords:** antibodies, native/denatured, egg albumin, casein, Alzheimer’s Disease, CSF, serum, gender, sequence/structural similarity, serpins

## Abstract

Alzheimer’s Disease (AD) is a multifactorial process. Amyloid plaque formation constitutes the main characteristic of the disease. Despite the identification of numerous factors associated with AD, the mechanism remains unclear in several aspects. Disturbances in intestinal and blood–brain barrier (BBB) penetration, observed in AD, may facilitate immunologic response to food-derived antigens. In the present study, antibodies against egg albumin, bovine-casein, and N-Glycolyl-Neuraminic acid (Neu5Gc) were measured in the cerebrospinal fluid (CSF) and serum of the patients using an enzyme-linked immunosorbent assay (ELISA). Zero anti-Neu5Gc and low concentrations of anti-casein antibodies were detected. Increased anti-native egg albumin antibodies were present in the serum of patients of all stages with 65% positivity (*p* < 0.001) in mild disease and a higher percentage in females (81.9%, *p* < 0.001). Lower serum positivity to anti-denatured egg albumin antibodies was observed, showing a gradual increase with severity and higher prevalence also in females. In the CSF, anti-native and anti-denatured egg albumin antibodies were mainly observed in severely ill patients with accumulative positivity to either antigen, reaching 61.8% in severe vs. 15% in mild disease (*p* < 0.001). Increased values were mainly observed in males. Anti-egg albumin antibodies may be implicated in the disease mechanism through sequence/structural similarity with human proteins, mainly serpins, and it would be worth consideration in further investigations and therapeutic strategies.

## 1. Introduction

Numerous studies have suggested the involvement of antibodies against food-derived antigens in the development and progression of several diseases, among which, bowel [1,2], autoimmune [3] and degenerative diseases [4], migraine [5], other neurological diseases [6,7,8], and mental disorders [9,10,11] have also been corelated with food-derived antigens.

Normally, IgA and IgM antibodies against food antigens may be detected in healthy people because of oral tolerance, a process related to the mucosal immune system’s ability to differentiate between pathogenic and non-pathogenic antigens, mostly described as immunological ignorance. However, dysregulation of this process may lead to increased concentrations of IgG as well as IgM food antigen-specific antibodies in the serum of certain individuals [12,13]. Cross-reaction of these antibodies with human proteins or peptides with increased sequence/structural identity or similarity with the food antigens (molecular mimicry) may be involved in the development or progression of related diseases [6,14,15].

Although humans consume a great variety of foods with thousands of proteins and other ingredients, a small number of foods and dietary components have been correlated with diseases. Among the most common food ingredients which are related to diseases is gliadin, a component of gluten proteins of wheat products, mostly related to intestinal disorders [1,2]. Gluten implication in other diseases such as multiple sclerosis (MS) [7,8], autism [16], and schizophrenia [17] has also been mentioned. However, it is not clear if there is a straight correlation or if the effect concerns the leaking of other factors to the serum because of the increased intestinal permeability caused by the reaction to gluten. Casein, the main milk protein, has also been related to MS, depression, and bipolar disorder, connecting neurological and mental disorders with dairy food consumption [8,10,12].

Specific dietary recommendations, based on food-antigen-specific antibodies, relieved the symptoms of diseases in some cases [8,18,19,20,21,22,23]. However, antibodies against specific food antigens are usually present only in a subgroup of patients in all related diseases, suggesting that such immune responses represent one of multiple contributing factors in disease onset or progression.

Alzheimer’s Disease (AD) is a multifactorial process. At the molecular level, the main characteristics of the disease include abnormal metabolism of the amyloid β precursor protein (AβPP), aggregation of the long length 42/43 amino acid amyloid-β peptide (42/43 Aβ peptide), and amyloid plaque formation. Hyperphosphorylation of tau peptide also occurs. In addition, oxidative stress and inflammation, as well as cholinergic neuron damage, are key pathological features involved in the disease process [24]. However, the etiology of AD is not clear yet, although several etiological factors have been proposed by different researchers. High pressure and cardiovascular disorders [25], diabetes [26] and hypercholesterolemia [27], brain traumatic injury [28], heavy metals, and other pollutants [29] through their involvement in oxidative stress [30] and inflammation [31], or not, have been correlated with increased risk for AD development. Quality of nutrition at middle age, 10–15 years before the onset of the symptoms of the disease, has also been related to AD onset [32], while the role of infectious factors, such as viruses [33], intestinal parasites [34], and intestinal microbiome [35], have recently garnered significant scientific interest.

Increased intestinal permeability [35], related or not to the above-mentioned intestinal disorders, is a common characteristic of patients with AD. This condition, along with the increased permeability of the blood–brain barrier (BBB), which also characterizes AD patients, may facilitate immunological response against food antigens, as was observed in other neurological disorders. Research investigating the cross-reaction of antibodies to specific food antigens with various purified human tissue antigens indicated a positive reaction between antibodies against egg, milk, wheat, and corn with myelin basic protein (MBP) or Aβ42 peptide, both related to amyloid plaque formation [12].

The presence of IgG antibodies against egg albumin have been found in human serum [36] although no correlation of such antibodies with neurological disorders has been mentioned until now. Mixed antibodies against egg and bovine antigens have displayed specificity toward human antigens, among which are proteins related to AD [12]. However, there was no evidence that antibodies against egg or bovine albumin are among these antibodies.

In the present study, three food antigens previously related or not related to neurologic disorders (egg albumin, casein, and Neu5Gc) were selected for the determination of antibodies against them in the cerebrospinal fluid (CSF) and serum of patients with AD and healthy individuals. Neu5Gc is a sialic acid present in animals but not in humans. Nevertheless, it can be incorporated into human glycoproteins, triggering immunologic response in some individuals, and has been correlated with autoimmune diseases and cancer [3,37]. The fact that the main protagonists of AD such as AβPP are glycoproteins raised the idea that an investigation into the probable involvement of anti-Neu5Gc antibodies is worth consideration.

The results indicated an increased concentration of antibodies against native and denatured egg albumin in the CSF of the majority of patients with severe Alzheimer’s Disease with a low presence in the CSF of patients with mild and moderate disease. In addition, an increased concentration of antibodies against egg albumin was present in the serum of AD patients of all stages.

An investigation of the sequence and structural similarity of egg albumin with human proteins indicated increased similarity with several proteins, some of which may be involved in the pathophysiology of the disease.

## 2. Materials and Methods

### 2.1. Materials

The biological samples were cerebrospinal fluid from 132 subjects, 13 cognitively normal and 119 patients with mild (57), moderate (14), and severe (48) AD, and serum from 56 healthy individuals and 60 patients with mild (20), moderate (20), and severe (20) Alzheimer’s Disease. All subgroups consisted almost equally of males and females. Their age varied between 52 and 90 years old, with an average age between 74 and 79 years and a median age between 73 and 79 years. Specific characteristics of the patients’ groups (age range, education, and MMSE range) are shown in Tables S1 and S2 of the Supplementary Materials.

The samples were kept at −80 °C until use. The samples were collected as part of the diagnostic tests of the patients and their use in research has the approval of the subjects or/and their families. All procedures are in accordance with the instructions of the Research Ethics Committee of I.H.U and A.U.TH., including safeguarding of personal data. The samples were provided by the “Hellenic Society of Alzheimer’s Disease and Related Disorders”, Alzheimer’s Disease Treatment Units “Agia Eleni” and “Agios Ioannis”, and the use of the samples was approved by the related bioethics committee (approval code: 74/28-01-2022).

Chemical and Biochemical Reagents: All chemical reagents were of analytical grade and were products of Merck (Darmstadt, Germany), Fluka (Buchs, Switzerland) and Riedel de Haen (Seelze, Germany). Microtiter 96-well, flat-bottom plates, precoated with highly hydrophilic microlon resin, were purchased from Greiner Bio-One (Kremsmünster, Austria). Egg albumin (2x crystalized) was purchased from Fluka AG, Switzerland, and casein (vitamin-free, BIOSYNTH) was purchased from Riedel-deHaen, Germany. Glycolylneuraminic acid (Neu5Gc) was a product of Sigma (St. Gallen, Switzerland). BSA, fraction V was purchased from AppliChem GmBH (Darmstadt, Germany). Peroxidase-conjugated anti-human IgG antibodies were purchased from AbD Serotec (Kidlington, UK). Human IgG antibodies were also purchased from AbD Serotec.

### 2.2. Methods

#### 2.2.1. Classification of AD Severity

The three stages of severity of AD were defined according to the results in the Mini-Mental State Examination (MMSE). Patients with MMSE > 20 had mild AD, patients with 10 < MMSE < 20 had moderate AD, and patients with MMSE < 10 had severe AD.

#### 2.2.2. Measurement of Anti-Egg Albumin, Anti-Casein, and Anti-Neu5Gc Antibodies [3]

For the determination of the antibodies, 96-well, flat-bottom plates, precoated with highly hydrophilic microlon resin, were used. The wells were coated with the appropriate antigen (egg albumin, casein, or Neu5Gc) by adding 100 μL of a solution containing 32 μg/mL of the antigen in 0.05 M carbonate buffer, pH 9.6. The solution was washed out after 16 h of incubation at room temperature. At the second stage, 200 μL of blocking solution containing 0.1% of blocking protein in Phosphate Buffer Salin, PBS (137 mM NaCl, 2.7 mM KCl, 10 mM Na_2_HPO_4_, and 2 mM KH_2_PO_4_), pH 7.2, was added to each well and was incubated for 2 h at room temperature. Bovine Serum Albumin (BSA) was used as the blocking protein for the determination of anti-egg albumin and anti-casein antibodies. For the determination of anti-Neu5Gc antibodies, egg albumin was used as the blocking protein because animal proteins may contain the Neu5Gc antigen. At the third stage, 100 μL of CSF diluted in PBS containing 0.05% Tween-20 and 0.1% of the blocking protein was added to each well and was incubated for 2 h at room temperature. Addition of the blocking protein at this stage aims to prevent co-determination of antibodies recognizing the blocking protein itself. Three different dilutions, 1:3, 1:6, and 1:15, were first used. Dilution at 1:6 was selected as the most appropriate for final application of the method. The 1:6 dilution was selected because it enabled a clear distinction between samples (absorptions between 0.400 and 1.800, within the optimal absorption scale of a microtiter plate reader) in the case of anti-egg albumin and anti-casein determination.

For the determination of anti-egg albumin antibodies in human serum, three different serum dilutions, 1:250, 1:500, and 1:1000, were first used. Dilution at 1:500 in PBS containing 0.05% Tween-80 (PBST) and 0.1% of the blocking protein was selected as the most appropriate for final application of the method.

After the aspiration of the supernatant and three consecutive washes with PBST and double distilled water, 100 μL of peroxidase-conjugated anti-human IgG (1: 10000) was added to each well. Following 1 h of incubation, the supernatant was rejected and after washing with PBST and double-distilled water, peroxidase substrate 3,3′,5,5′-Tetramethylbenzidine (TMB) was added to the wells. The colored product produced was measured with an ELISA reader at 630 nm. All samples were measured in duplicate. In all cases, the difference between two measurements of the same sample was less than 10%. In each plate, at the coating stage, two pairs of 8 wells were coated in duplicate with different concentrations of human IgG antibodies in 0.05 M carbonate buffer, pH 9.6, and were used for the construction of a standard curve. For verification of the method, the determination was repeated in some positive samples with and without addition of a free soluble antigen in the sample dilution mixture to result in the expected reduction in the measured value in the presence of free antigen, as further proof that the measurement was due to antibodies recognizing the bound antigen.

All experiments were performed in triplicate, and the average value is included in the results.

For the determination of antibodies against denatured egg albumin, heat denaturation of native egg albumin was performed. An equal mixture of heat-denatured egg albumin under different heating conditions was used. For the preparation of the mixture, 10 mg/mL egg albumin in 25 mM Tris-HCl pH 8.5 was heated at 37 °C for 4 h, 37 °C for 24 h, 50 °C for 4 h, 50 °C for 24 h, and 80 °C for 4 h. Figure S1 shows the electrophoretic analysis of the heat-denatured egg albumin and their recognition by anti-denatured egg albumin anti-serum. The different temperature conditions/duration were applied to achieve all probable stages of denaturation, from simple conformation changes to protein polymerization [38].

The same procedure was used for the preparation of denatured casein.

#### 2.2.3. Statistical Analysis

The Shapiro–Wilk W test [39,40] was used to check the normality of distribution of the values of antibodies in the subgroups of patients with AD. The Mann–Whitney nonparametric test for independent samples was used to evaluate the statistical significance of differences in the concentrations of antibodies between groups [39,41]. The Chi-square test [42] and Fisher’s exact test (conditional, two-tailed) [43] was used to evaluate the statistical significance of the difference in percentage of positives between two groups.

#### 2.2.4. Protein Similarity Search

For the protein similarity search, the 386 amino acid sequence of ovalbumin (Gallus gallus—chicken), Uniprot entry P01012, was used. The search was performed using the basic BLASTp tool of Uniprot and the UniProtKB database was selected. The search was restricted by taxonomy to [Homo Sapiens 9606]. The target hits were set to 1000. The rest of the parameters were kept as initially set by the program (e-threashold:10, matrix: auto-BLOSUM62, filter: none, gapped: yes, HSPs per Hit: all). For 3D structure comparison, the RCSB pairwise structure alignment tool was used.

## 3. Results

Preliminary results were first obtained by the identification of antibodies against Neu5Gc, native casein, and native egg albumin in the cerebrospinal fluid (CSF) of 13 healthy individuals, 17 AD patients with mild disease, 14 patients with intermediate, and 14 with severe AD. The preliminary results indicated no anti-Neu5Gc antibodies in all stages of the disease. However, increased concentrations of antibodies against egg albumin and, to less extent, against bovine casein were found in the CSF of patients with severe disease.

Το confirm the preliminary results and also evaluate the existence of antibodies against denatured egg albumin, a second independent experiment was performed using a second set of 74 CSF samples of mild (40) and severe (34) disease. The characteristics of the patient groups (age, education, and MMSE) are presented in Table S1 of the Supplementary Materials.

**Figure 1:**
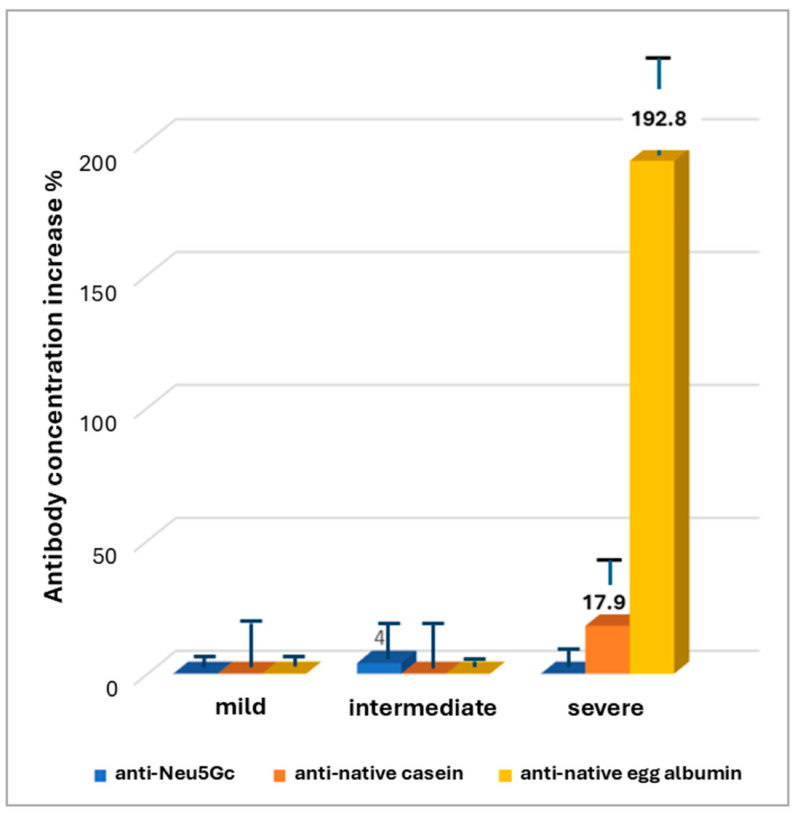
Increase in mean antibody concentration compared to the group of apparently healthy subjects.

The results confirmed the previous observation for anti-native egg albumin antibodies, indicating a 192.8 ± 36.2% increase in mean concentration of antibodies against native egg albumin in patients with severe AD, compared to healthy individuals (Figure 1). A lower, not statistically significant increase of 17.9 ± 24.3% was observed in the mean concentration of antibodies against native bovine casein, in severe disease, too. No increase or a slight decrease was observed in the mean concentration of antibodies against both antigens in patients with mild and intermediate disease. The increase in mean concentration of anti-egg albumin antibodies in severe compared to mild disease was substantial and statistically significant according to the Mann–Whitney test (U = 187.5, Z = −5.07201, *p* < 0.00001). However, a low number of samples with increased concentrations (positive samples) were observed in mild disease, as well.

**Figure 2:**
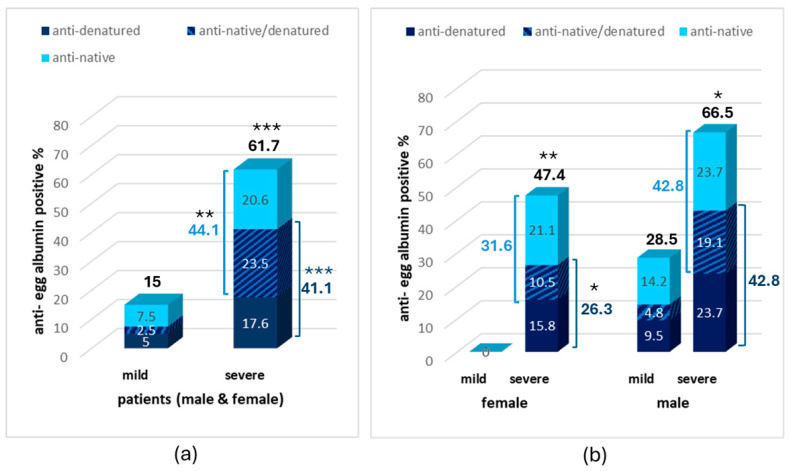
Percentage of anti-egg albumin positive samples in the CSF of patients with mild and severe AD in the whole sample (**a**) and in the male and female subgroups (**b**). Statistically significant difference with *** *p* < 0.001, ** *p* < 0.01, * *p* < 0.05 (Fisher’s Exact test, two-tailed) compared to the mild AD group.

Concerning positivity, if all samples with an antibody concentration higher than the mean concentration of healthy subsects + 2SD are considered as positive, a 44.1% positivity in antibodies against native egg albumin was observed in severe AD vs. 10% in patients with mild AD (*p* < 0.01, Figure 2). 

The identification of antibodies against denatured egg albumin revealed an increased percentage of patients with anti-denatured egg albumin antibodies also in severe AD (41.1%) compared to mild AD (7.5%), increasing the percentage of antibody-positive samples against one or both egg albumin forms to 61.7% in severe compared to 15% in mild disease (*p* < 0.001, Figure 2a).

Most samples with severe AD (23.5%) were positive for both the anti-native and anti-denatured egg albumin antibodies with 20.6% and 17.6%, respectively, being positive to antibodies against only the anti-native or anti-denatured form. On the contrary, only 2.5% of patients with mild disease were positive to both antibodies, with 7.5% and 5%, respectively, being positive to antibodies against only the anti-native or anti-denatured form.

Interestingly, a higher increase in anti-egg albumin antibodies was observed in the CSF of male individuals compared to females (Figure 2b). The percentage of positives to at least one antibody in the CSF of males with severe AD was 66.5% vs. 47.4% in females. The same was observed for positives to each one of the antibodies (42.8% vs. 31.6% for anti-native or 42.8% vs. 26.3% for anti-denatured form). Most interestingly, no positive samples were observed in females with mild AD while 28.5% of total positive samples were observed in males with mild AD.

The question eventually raised following the above observation was if the increase in the CSF follows an analogue increase in antibodies in the serum.

For this reason, anti-egg albumin antibodies were measured in the serum of 57 healthy individuals and in the serum of 60 patients with mild (20), intermediate (20), and severe (20) disease. The characteristics of the patient groups (age range, education, and MMSE range) are presented in Table S2 of the Supplementary Materials.

**Figure 3:**
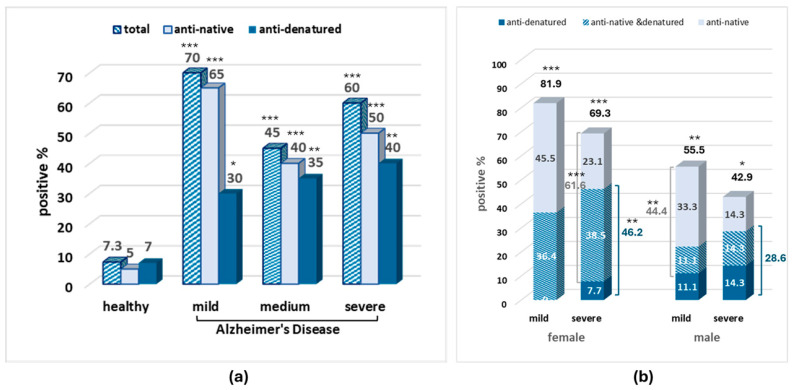
Percentage of anti-egg albumin-positive samples in the serum of patients with mild and severe AD in the whole sample (**a**) and in the male and female subgroups (**b**). Statistically significant difference with *** *p* < 0.001, ** *p* < 0.01, * *p* < 0.05 (Fisher’s Exact test) compared to the healthy–all (male and female) group.

Increased serum positivity was observed in patients with AD of all stages varying between 30% and 81.9% according to the recognized egg albumin form, the sex, and the stage of the disease vs. 5–7.3% in healthy individuals (Figure 3).

Interestingly, in the serum samples, the amount of total and anti-native egg albumin antibodies is higher in mild than in severe AD in both males and females, while the amount of anti-denatured albumin antibodies seems to increase with severity. The second interesting observation concerns sex differences, where, in contrast to the CSF, the higher antibody positivity in serum samples was observed in females in both severity stages.

Since, specific mechanisms affecting the development of antibodies against food antigens may affect the concentration of more antibodies in human serum, the antibodies against bovine casein were also measured in the serum of Alzheimer’s patients and healthy individuals.

**Figure 4:**
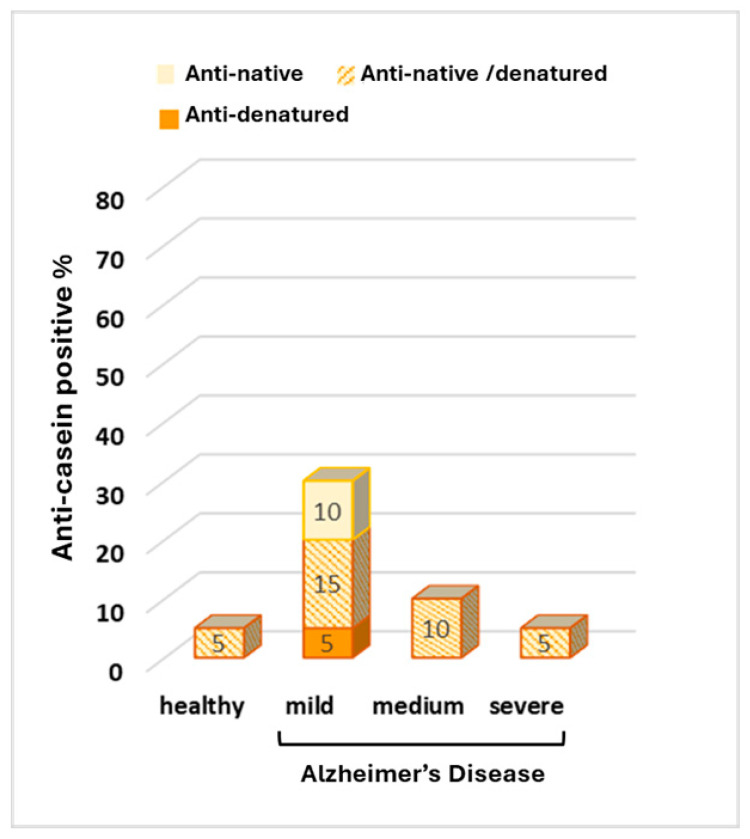
Percentage of anti-casein antibodies in the serum of healthy individuals and in the serum of patients with mild, medium, and severe Alzheimer’s Disease.

An increase in total anti-casein-positive samples compared to healthy individuals was observed in the serum of patients with mild and intermediate AD (30% and 10%, respectively, vs. 5% in healthy subjects). However, the observed differences were not statistically significant (Figure 4).

As molecular mimicry is among the most prominent mechanisms correlating increased antibodies against food antigens and disease development, the sequence similarity of egg albumin with human proteins was searched using the Uniprot BLASTp tool (Table 1).

According to the results, 35 human serine protease inhibitors (serpins) and 3 other human proteins were found to have sequence identity with egg albumin ranging between 21 and 41.4%, thirty-five of them with very low e-values ranging between 4.8 × 10^−9^ and 1.2 × 10^−52^.

However, the sequence identity alone does not justify a probable implication of egg albumin or anti-egg albumin antibodies in the mechanism related to the function of these molecules. Especially when the native form of the antigen is considered, a combination of structural and sequence identity or similarity together with the placement of the homologue parts at the surface of the molecules is mandatory to ensure similarity in function or antigenic reaction.

**Figure 5:**
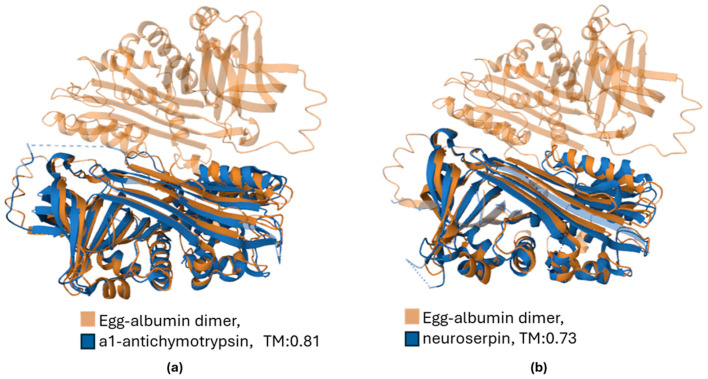
Three-dimentional structure alignment of egg albumin (ovalbumin, PDB ID: 1OVA) with (**a**) a1-antichymotrypsin (serpin A3, PDB ID: 1QMN) and (**b**) neuroserpin (serpin I1, PDB ID: 3F02). The Template Modeling score (TM), ranging from 0 to 1, obtains high values, indicating a relatively accurate mach.

The 3D structure similarity between egg albumin and two representative serpins, the a1-antichymotrypsin (serpin A3, sequence identity 30%) and neuroserpin (serpin I1, sequence identity 29.2%) is shown in Figure 5.

**Figure 6:**
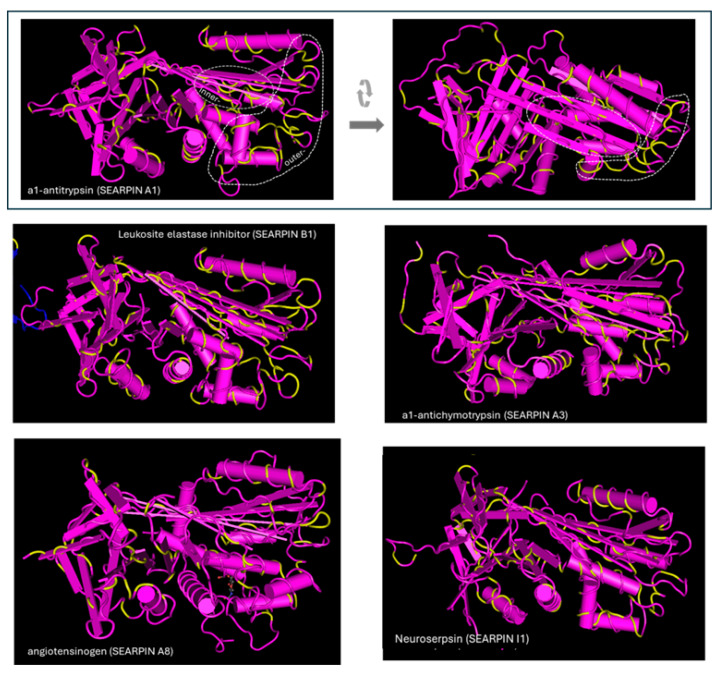
Amino acid identity of serpins with egg albumin. The identical amino acids are shown in yellow. Identical amino acids are found at outer areas and in inner regions, which can be exposed and trigger immunologic response during denaturation. The structures were obtained from the Protein Data Bank and have the following PDB IDs: a1-antitrypsin: 3NE4, neuroserpin: 3F5N, a1-antichymotrypsin: 1QMN, serpin B1: 4GA7, angiotensinogen: 5M3Y.

The location of amino acids of representative serpins with sequence identity with egg albumin is presented on the 3D structures of the serpins in Figure 6. As shown in the pictures, the accumulation of identical amino acids is found in some outer areas. Other accumulations are found in inner regions. Denaturation is a prerequisite for exposure and triggering immunologic response by the latter.

## 4. Discussion

### 4.1. Main Observations

According to the results, increased antibodies against egg albumin were observed in the CSF of a great proportion of patients with severe Alzheimer’s Disease. Increased antibodies against native egg albumin were observed in 44.1% of severely ill AD patients, a frequency about 4.5-fold higher than that occurring in patients with mild disease (Figure 2). No positive samples were found in apparently healthy individuals. If the proportion of the patients found to be positive to anti-denatured egg albumin antibodies is added, the average percentage of anti-egg albumin-positive samples in patients with severe AD reaches 61.7% in the mixed population and 66.5% in male patients. This constitutes a markedly elevated percentage, compared to healthy controls and patients with mild AD, implying a correlation between positivity and the disease (Figure 2).

CSF antibodies can be of serum origin or can be produced within the Central Nervous System (CNS) [44]. The presence of anti-egg albumin in the serum of patients of all stages of AD and in the CSF of severely ill patients could justify both origins. However, the different patterns of positive samples among AD stages, gender subgroups, and nature of the recognized egg albumin in the serum and CSF implies a more complicated mechanism than passive diffusion of serum antibodies to the CSF (Figure 2 and Figure 3).

Increased anti-native egg albumin antibodies are present in the serum of the great majority of patients with mild AD (positivity 65%), with a higher percentage among females (81.9%). The positivity remains high, although slightly reduced in the severely ill patients. However, a low positivity in anti-native egg albumin antibodies is present in the CSF of patients with mild AD, with zero positivity in the female subgroup. The anti-egg albumin positivity at the CSF is gradually increased with severity, with higher positivity and earlier appearance in the male subgroup. This corresponds to the opposite distribution of positivity in CSF between gender and severity subgroups compared to serum (Figure 2 and Figure 3).

A characteristic worth mentioning and common in serum and CSF samples is the positivity to anti-denatured egg albumin, gradually increasing with severity in both gender subgroups (Figure 2 and Figure 3).

Increased concentrations of antibodies against food-derived antigens like egg albumin presuppose an intestinal or immunologic disturbance such as a leaky intestine. Moreover, increased blood–brain barrier (BBB) permeability may contribute to the high antibody concentration in cerebrospinal fluid. Both intestinal and BBB disturbances are common in AD patients [12]. Immune system impairment may also characterize some of the patients as it is a common characteristic of aging [45]. However, the observed increased positivity against egg albumin cannot be a general result of the leaky intestine and increased BBB permeability, since the same increase was not observed in the other food-derived antigens which were tested, Neu5Gc and casein. The results indicate the existence of a mechanism where specific characteristics of egg albumin are involved (Figure 1, Figure 3 and Figure 4). The increase in positivity against denatured egg albumin with disease severity implies (a) the existence and gradual enhancement of a factor which causes the probable exposure of internal areas of egg albumin, producing conformations present in the denatured protein and in protein aggregates or (b) gradual progress in intestinal permeability impairment, which enables the passage and immunologic reaction of the denatured egg albumin molecules. A gradual impairment of intestinal permeability by involvement of mechanisms enabling the passage of larger molecules could not alone explain the results. It could justify the penetration of denatured egg albumin polymers and the increase in serum anti-denatured albumin antibodies but could not explain the decrease in serum anti-native albumin antibodies. This could only be explained by the gradual presence of progressively stronger denaturing conditions or conditions favoring the polymerization of denatured ovalbumin. Protein denaturation and exposure of internal areas of the molecule is a naturally occurring event in the food digestion process of healthy individuals, mainly achieved due to the acidic conditions of the stomach and the effect of bile salts. The differentiations of the gut environment in AD patients are mainly related to changes in gut microbiota and include (a) leaking intestine; (b) oxidative and inflammatory conditions [46]; (c) the presence of Aβ aggregates induced or produced by intestinal microbes; and (d) the presence of substances closely related to Aβ aggregates, such as acrolein, capable of inducing protein polymerization [47,48]. Polymerization is a natural property of all members of the serpin family, although it is observed in many proteins. In conditions favoring relaxed conformation of the serpin molecules, intramolecular interactions can occur, leading to inactive serpin dimers or trimers, a procedure involved in serpin activity control [49]. Egg albumin, although a serpin, does not follow the conformation changes occurring in classic serpin polymerization. However, it polymerizes under about the same conditions and may affect other proteins’ polymerization [50]. In addition to extreme pH conditions, the increased surfactants secreted in Alzheimer’s Disease may facilitate relaxation and polymerization, a condition which may favor Aβ aggregation, as well [51,52,53,54], while oxidative conditions may facilitate the stabilization of denatured and polymerized proteins [55]. In Alzheimer’s Disease, bacterial dysbiosis, the main cause of impaired intestinal permeability [56] is a common and gradually progressive characteristic affecting both the colon and small intestine [57,58], with colon dysfunction detected in the first stages of the disease [59]. The sex differences known to exist in intestinal microbiota [60,61], oxidative conditions [62,63,64], and intestinal permeability, as well as the increased male susceptibility in BBB disfunction [65] may contribute to the sex differences observed anti-egg albumin antibodies in the serum and CSF. Although events of the disease may justify the observed results, experimental work is needed to further evaluate the probable correlation.

In general, when conditions like leaky intestine exist, food antigens can cause disorders through at least three different ways, both based on similarity of the molecule with human proteins: (a) production of antibodies, which may cross-react with the human proteins, usually diminishing the concentration of these proteins, disrupting the mechanism served by them, and triggering inflammatory responses [66]; (b) direct involvement in a mechanism, since molecular mimicry may enable appropriate interactions [67], and worth mentioning is that, although egg albumin is an inactive serpin with no serine protease inhibitory action, certain hydrolysates, such as ovokinim, have been found to act as ACE inhibitors [68]; and (c) interaction with the target molecule of the homologue protein in an unsuccessful manner, preventing the successful interaction of the homologue protein.

Homology with human proteins is a prerequisite for any of the probabilities mentioned above although further experiments are needed to explore if any of these probabilities are applied in the case of egg albumin and the antibodies recognizing it in patients with Alzheimer’s Disease.

### 4.2. Homology with Human Proteins

BLAST research (Table 1) revealed 21–42% similarity of ovalbumin with 35 human serine protease inhibitors (serpins) and with 3 non-serpin proteins: the Serpin-like minor histocompatibility protein HMSD, the phosphatidylinositol 5-phosphate 4-kinase type-2 gamma (PIP4K2C), and the N-acylglucosamine 2-epimerase (RENBP).

As shown in the table, many of these molecules are related to the nervous system physiology or AD-linked mechanisms and relevant disorders [69,70,71,72,73].

### 4.3. Intestinal Permeability and Inflammation

The serpin B1 sharing 38.8% identity with ovalbumin is a serpin secreted by the neutrophils [74], responsible for cell protection during inflammation, a common disorder in AD, with an involvement in the maintenance of endothelial cell junction integrity [75,76,77,78]. It can inhibit proteases secreted during inflammation. Among the above proteases, chymase can enhance epithelial permeability by causing redistribution of the tight junction proteins ZO-1 and occludin [77,78]. Serpines A2 and A3 with 28.1% and 30.0% identity, respectively, also act as inhibitors of cathepsin and chymase proteases that affect epithelial permeability. The intestinal equilibrium between proteases and serpins is of great importance for intestinal health and proper function. Antibodies recognizing serpins could impair serpin metabolism, affecting this equilibrium [79,80].

### 4.4. Blood–Brain Barrier

The serpin A8 (identity 21%), known as angiotensin (AGT) is present both in circulation and in the brain, with lower levels of circulating AGT present in AD patients compared to in the healthy population [81]. AGT, as well as its active metabolite, angiotensin II (AngII), are also involved in the control of cerebral blood flow, memory retention, neuronal regeneration, and BBB permeability [70,82] and may have a role in cognition and brain health [71,83].

### 4.5. CNS

The proteolytic mechanism is crucial in AD development. Several proteases, among which a-, β-, and γ-secretase as well as proteases involved in their maturation and clearance, are closely connected to beta-amyloid production and degradation. Consequently, the balance of proteases and protease inhibitors is of great importance [24]. Although the AD-related secretases are aspartate proteases and thus not inhibited by serpins, their maturation and activity are regulated by other serin proteases like fucin and thus may be indirectly affected by serpins [84]. Serine proteases (plasmin, akylpeptide hydrolase, and myelin basic protein) with different impacts in natural Aβ and fibril degradation are among the Aβ peptide-clearing enzymes, although they constitute a minority of the proteolytic enzymes involved [24,85]. Serpins which have been related with Alzheimer’s Dis-ease and with the brain events which accompany the disorder are serpin A1 (a1-antitrypsin, identity 30%), A3 (a1-antichymotrypsin) [69,86,87,88,89,90] and B1, serpin I1 (neuroserpin, identity 29.2%) [91,92,93,94,95,96,97,98,99], serpin E1 (identity 27.5%), F1 (identity 22.6%) [100], and serpin A8 (AGT) [101]. An increase in serpin concentration is observed in most cases [69,86,95], although a decrease [94] has been observed in some cases, depending on disease stage or presence of other protease inhibitors [94,95]. Serpins in CNS have been associated with either a negative or neuroprotective role [92,93]. It is not clear if upregulation, when observed, is part of the etiology of the disease or if it is a response to the elevated protease activity, which increases to counteract the accumulation of disease-related protein aggregates [87], a response to the reduction in other protease inhibitors [94,95], or a response to other dysregulating processes. It would be worth exploring the role of antibodies recognizing serpins in this process.

A more detailed study on the involvement of serpins with high sequence identity with egg albumin in AD pathophysiology is provided in Supplementary Text S1. 

### 4.6. Aspects on Prevention and Therapeutic Management

Increased antibodies against egg albumin are present in the serum and CSF of a great portion of patients with Alzheimer’s Disease, and sequence and structural similarity suggest probable interactions with disease-related proteins that may affect normal function. So, the removal of egg whites from the diet of antibody-positive patients could probably be worth considering as a method of therapeutic management.

At first glance, this concept seems to be in contradiction with projects supporting egg consumption as a means of alleviating disease symptoms [102,103,104,105,106]. There is a number of studies showing a decrease in AD prevalence related to egg consumption [102,103,104,105,106]. A low improvement in the female group or maintenance of cognitive function was observed in egg-consuming AD patients over a four-year period [106]. These studies concern whole egg consumption and researchers often hypothesize that beneficial ingredients such as choline [102] belong to the yolk. Moreover, there are studies indicating no effect in a studied population group, such as a study in Spain where no effect was found in people following the Mediterranean diet (MD), and a borderline negative correlation was found between egg consumption and dementia in people with low adherence to the MD [103]. Probably, the existence of beneficial effects or the differentiation in the appropriate egg number is related to the lack of specific substances, most probably of non-protein nature, from the daily diet of the subjects.

If egg albumin can cause opposite effects in some subjects by triggering immune response, the exclusion of egg whites from the diet of positive subjects could augment the beneficial effects of the yolk in relation to AD delay and improvement, and it is worth testing. Under the same concept, better results could be obtained if egg white was avoided as a protein source by egg albumin antibody-positive patients in efforts to make cognitive improvements with increased protein consumption strategies [107].

## 5. Conclusions

The present study indicated increased levels of antibodies recognizing egg albumin in the serum and CSF of a great percentage of patients with AD, which correlates immune response to egg albumin with Alzheimer’s Disease. The results indicated a disease-severity-linked increase in anti-denatured egg albumin antibodies in the serum of AD patients in contrast to the nearly constant presence of serum anti-native egg albumin antibodies in all disease stages. In the CSF, positivity to antibodies against both egg albumin forms substantially increased with severity when in the low presence of antibodies in the CSF of males and zero presence in the CSF of females with mild disease. Sex differences are further extended as higher serum positivity is observed in females and higher CSF positivity in males.

The specific characteristics of the immune response may be related to specific events during disease development related to the gradual changes in intestinal microbiota, intestinal, and BBB permeability and to the sex differences observed in these disturbances.

Sequence and structural identity/similarity of egg albumin with human proteins, mainly serpins involved from barrier’s permeability to Aβ peptide metabolism may support the probable participation of antibodies to disease development. Further experiments are needed to explore the probability of involvement of anti-egg albumin antibodies in AD pathophysiology. In any case, positivity to anti-egg albumin antibodies is observed in a remarkable proportion of AD patients, and it would be worth taking this into account in the therapeutic strategies of the disease.

## Figures and Tables

**Table 1:** Results of protein similarity search between egg albumin and human proteins using the Uniprot BLASTp tool. One of the hits concerning the same molecule is referred to in this table.

BLAST Ovalbumin—Homo Sapiens			
Protein	Description *	Identity	e-Value
**Serpins**			
A1	Serine protease inhibitor (serpin) A1: α1-antitrypsin, trypsin and chymotrypsin inhibitor, ER and extracellular, anti-coagulant, lung protection	30.0%	1.2 × 10^−52^
A2	α1-antitrypsin-like (intracellular glycoprotein), inhibition of cathepsin and mast cell chymase (angiotensin I to II converting enzymes)	28.1%	1.8 × 10^−42^
A3	α1-antichymotrypsin (cell-growth inhibiting gene), intra-/extracellular, inhibition of cathepsin and mast cell chymase (angiotensin I to II converting enzymes), inflammation, lipid metabolism, extracellular matrix remodeling, Alzheimer’s Disease.	30.0%	3.6 × 10^−52^
A4	Kallistatin (Kallikrein inhibitor, extracellular glycoprotein, expressed in leukocytes, liver, esophagus, and brain)	27.9%	5.5 × 10^−38^
A5	Serum, urine and seminal serpin, heparin-dependent, anti-coagulant	29.2%	5.7 × 10^−47^
A6	Extracellular serpin, steroid binding	26.9%	2.6 × 10^−40^
A7	Serpin, thyroid hormone serum transporter	25.3%	4.3 × 10^−36^
A8	Angiotensin (AGT). Regulation of blood pressure and electrolyte homeostasis. AGT-2: Vasoconstriction (AGT2), cardiac contractility, and heart rate regulation through its action on the sympathetic nervous system. AGT-3: Aldosterone synthesis and release. AGT 1-7: Vasodilator, antidiuretic effects, antithrombotic through MAS1 (mitochondrial assembly 1)-mediated release of nitric oxide from platelets. Sodium channel regulation, kidney development, vascular remodeling, LDL (Low-Density Lipoprotein) remodeling, associative learning. Involvement in long-term neuronal synaptic plasticity, regulation of transmission of nerve impulse, regulation of norepinephrine secretion, response to estradiol, regulation of protein import into nucleus, nitric oxide-mediated signal transduction, phospholipase C-activating G protein-coupled receptor signaling pathway, RK1 (Extracellular Signal-Regulated Kinase 1) and ERK2 (Extracellular Signal-Regulated Kinase 2) cascade, stress-activated MAPK (mitogen-activated protein kinase) cascade, G protein-coupled receptor signaling Negative regulation of angiogenesis, cell growth, tissue remodeling, gene expression, MAP kinase activity, neurotrophin TRK (Tropomyosin Receptor Kinase) receptor signaling Positive regulation of activation of Janus kinase activity, CoA-transferase activity, NAD(P)H oxidase activity, peptidyl-tyrosine phosphorylation, phosphatidylinositol 3-kinase signaling, protein tyrosine kinase activity, superoxide anion generation, reactive oxygen species metabolic process fibroblast proliferation, endothelial cell migration, positive regulation of vascular-associated smooth muscle cell migration, epidermal growth factor receptor signaling, extrinsic apoptotic signaling pathway, inflammatory response, cytokine production, macrophage derived foam cell differentiation, insulin receptor signaling, membrane hyperpolarization, cytosolic calcium ion concentration, extracellular matrix constituent secretion, L-arginine/L-lysine import across plasma membrane, NF-kappaB (Nuclear factor kappa-light-chain-enhancer of activated B cells) transcription factor activity, nitric oxide biosynthetic process	21.0%	4.8 × 10^−9^
A9	Extracellular	29.5%	2.9 × 10^−47^
A10	Serine protease inhibitor, endoplasmic reticulum and extracellular, anticoagulant, heparin-activated, liver regeneration	25.7%	2.7 × 10^−37^
A11	Serine Protease Inhibitor, extracellular	27.7%	1.8 × 10^−36^
B1	Extra-/intracellular, neutrophil serine protease inhibitor, cell protection during inflammation, regulates activity of elastase, cathepsin G, proteinase 3, chymase, chymotrypsin, kallikrein 3, caspase 1,4,5, Granzyme H (GZMH), promotes the proliferation of beta cells	38.8%	3.9 × 10^−87^
B2	Negative regulation apoptosis, fibrinolysis, extra-/intracellular, plasma membrane	36.9%	8.8 × 10^−83^
B3	SCCA-1 (Squamous Cell Carcinoma Antigen 1), intra-/extracellular, Serine/Cysteine Protease Inhibitor, autocrine/paracrine signal, positive regulation of cell migration and proliferation, anti-apoptotic, modulate the host immune response against tumor cells	41.4%	3.8 × 10^−104^
B4	SCCA-2, modulate the host immune response against tumor cells. Intra-/extracellular	40.9%	1.3 × 10^−99^
B5	Tumor suppressor	30.4%	6.8 × 10^−68^
B6	Probable involvement in the regulation of serine proteinases present in the brain or extravasated from the blood. Inhibitor of cathepsin G, kallikrein-8, and thrombin. Related to hearing loss.	35.5%	2.0 × 10^−83^
B7	Might function as an inhibitor of Lys-specific proteases. Might influence the maturation of megakaryocytes via its action as a serpin. Intra-/extracellular, mesangial cells, epidermis, positive regulation of collagen biosynthesis, platelet-derived growth factor, transforming growth factor β1, mesangial cell proliferation.	34.7%	8.0 × 10^−81^
B8	Important role in epithelial desmosome-mediated cell–cell adhesion. Intra-/extracellular. Cytoplasmic antiproteinase 2.	33.7%	2.9 × 10^−76^
B10	May play a role in the regulation of protease activities during hematopoiesis and apoptosis induced by TNF. May regulate protease activities in the cytoplasm and in the nucleus.	40.3%	8.9 × 10^−99^
B11	Probable loss of serine protease inhibitory action due to mutation.	38.8%	2.4 × 10^−100^
B12	Inhibits trypsin and plasmin, but not thrombin, coagulation factor Xa, or urokinase-type plasminogen activator. May play a role in cell differentiation. Intra-/ extracellular.	37.3%	1.3 × 10^−88^
B13	May play a role in the proliferation or differentiation of keratinocytes. Serine and cysteine protease inhibitor. Intra-/extracellular.	39.2%	2.8 × 10^−90^
C1	Antithrombin III. Most important serine protease inhibitor in plasma that regulates the blood coagulation cascade. Inhibits thrombin, IXa, Xa, Xia	32.5%	9.7 × 10^−57^
D1	Thrombin inhibitor activated by the glycosaminoglycans, heparin, or dermatan sulfate. In the presence of the latter, HC-II becomes the predominant thrombin inhibitor in place of antithrombin III (AT-III). Also inhibits chymotrypsin, but in a glycosaminoglycan-independent manner. Peptides at the N-terminal of HC-II have chemotactic activity for both monocytes and neutrophils	29.6%	1.8 × 10^−49^
E1	PAI-1, inhibitor of tissue-type plasminogen activator (PLAT) and urokinase-type plasminogen activator (PLAU). Cell migration/adhesion. Role in lugs, keratinocyte migration, odontogenesis.	27.5%	1.2 × 10^−43^
E2	Glia-derived nexin	30.6%	1.4 × 10^−46^
E3	Extracellular serine protease inhibitor	25.6%	2.1 × 10^−31^
F1	Pigment epithelium-derived factor. Neurotrophic protein; induces extensive neuronal differentiation in retinoblastoma cells. Potent inhibitor of angiogenesis. Probably no serine protease inhibitory action. Intra-/extracellular. Aging, short-term memory, cellular response to glycose, retinoic acid, dexamethasone, cobalt ion, arsenic containing substances, acidic pH, peptide. Positive regulation of neurogenesis, neuron projection development. Negative regulation of neuron death and inflammation, ovulation, prostate gland, and kidney development.	22.6%	6.8 × 10^−24^
F2	a2-antiplasmin, extracellular	26.0%	1.4 × 10^−31^
G1	Plasma protease C1 inhibitor. Activation of the C1 complex. Regulates complement activation, blood coagulation, fibrinolysis, and the generation of kinins. Inhibits FXIIa, chymotrypsin, and kallikrein. Role in innate immune response and aging.	24.6%	2.1 × 10^−21^
H1	Collagen biosynthesis, protein maturation.	26.7%	1.2 × 10^−41^
I1	Neuroserpin. Serine protease inhibitor that inhibits plasminogen activators and plasmin but not thrombin. May be involved in the formation or reorganization of synaptic connections as well as in synaptic plasticity in the adult nervous system. May protect neurons from cell damage by tissue-type plasminogen activator (probable)	29.2%	1.5 × 10^−61^
I2	Myoepithelium-derived serine protease inhibitor. Pancpin. Cell adhesion. Extracellular.	32.1%	8.5 × 10^−63^
**Other** **proteins**			
HMSD	Histocompatibility Minor Serpin Domain Containing (HMSD) protein: serpin-like protein, probable serine protease inhibitor. Activation of immune response. Extracellular.	25.8%	1.2 × 10^−10^
PIP4K2C	Phosphatidylinositol 5-phosphate 4-kinase type-2 gamma. Phosphatidylinositol phosphate biosynthetic process, negative regulation of insulin receptor signaling, positive regulation of autophagosome assembly.	26.2%	2.7
RENBP	Renin-binding protein (RENBP): N-acylglucosamine 2-epimerase. Catalyzes the interconversion of N-acetylglucosamine to N-acetylmannosamine. Binds to renin forming a protein complex called high-molecular-weight (HMW) renin and inhibits renin activity. Involved in the N-glycolylneuraminic acid (Neu5Gc) degradation pathway.	29.7%	5.2

* Corresponds to information provided by Uniprot for the entry with high identity exported using BLAST.

## Data Availability

A set including all data used for the production of the histograms presented at Figure 2 and Figure 3 and the relative statistical analysis results were uploaded. In addition the uncropped pictures of electrophoresis gels and western blots of Supplementary Figure S1 were also uploaded as a minimal data set. Any additional data can be made available by the authors on request.

## Supplementary Materials

The following supporting information can be downloaded at https://www.mdpi.com/article/10.3390/biom15081085/s1,: Table S1: Characteristics of the AD patients providing the CSF samples for the results presented at Figure 2; Table S2: Characteristics of the AD patients providing serum samples for the results presented at Figure 3; Figure S1: Denatured egg-albumin transfer and immunoblot using anti-serum of healthy and anti-denatured egg-albumin positive AD patients, after native and SDS electrophoresis. High MW egg-albumin bands, probable polymerization products, are detected and immunoblot-stained.; Text S1: “Involvement of serpins with high sequence identity with egg-albumin in AD pathophysiology”.

## Abbreviations

The following abbreviations are used in this manuscript:

AD	Alzheimer’s Disease
BBB BLAST BLASTp	Blood–Brain Barrier Basic Local Alignment Search Tool Basic Local Alignment Search Tool for proteins
MBP	Myelin Basic Protein
CSF	Cerebrospinal Fluid
Neu5Gc	Glycolylneuraminic Acid
I.H.U.	International Hellenic University
A.U.TH.	Aristotle University of Thessaloniki
MMSE	Mini-Mental State Examination
AGT	Angiotensin
ERK3	Extracellular Signal-Regulated Kinase 3
MAPK	Mitogen-Activated Protein Kinase
TRK	Tyrosine Receptor Kinase
NAD(P)H	Nicotinamide Adenine Dinucleotide Phosphate
NF-KappaB	Nuclear Factor-Kappa B
ER	Endoplasmic Reticulum
GZMH	Granzyme H
SCCA	Squamous Cell Carcinoma Antigen
HMSD	Histocompatibility Minor Serpin Domain Containing
HMW	High Molecular Weight
PIP4K2C	Phosphatidylinositol 5-Phosphate 4-Kinase Type-2 Gamma
RENBP	Renin-Binding Protein
PAI-1	Plasminogen Activator Inhibitor 1
PLAT	Tissue-Type Plasminogen Activator
PLAU	Urokinase-Type Plasminogen Activator
SEC	Serpin-Enzyme Complex
CNS	Central Nervous System
Aβ	Amyloid-Beta
AβPP	Amyloid-Beta Precursor Protein
AGTR	Angiotensin Receptor
MD	Mediterranean Diet
BSA	Bovine Serum Albumin
SDS	Sodium Dodecylsulfate
MW	Molecular Weight
