# Supplementary material for: Increased Concentration of Anti-Egg Albumin Antibodies in Cerebrospinal Fluid and Serum of Patients with Alzheimer’s Disease—Discussion on Human Serpins’ Similarity and Probable Involvement in the Disease Mechanism
Source: Biomolecules. 2025 Jul 27;15(8):1085. doi: 10.3390/biom15081085 (PMC12383860; doi:10.3390/biom15081085)
Supplement: Supplementary file 1 [file biomolecules-15-01085-s001.zip › Supplementary Figure S1 last.pdf]

Supplementary Figure S1: Denatured egg-albumin transfer and immunoblot using anti-serum of healthy and anti-denatured egg-albumin positive AD patients, after native and SDS electrophoresis. High MW egg-albumin bands, probable polymerization products, are detected and immunoblot-stained.

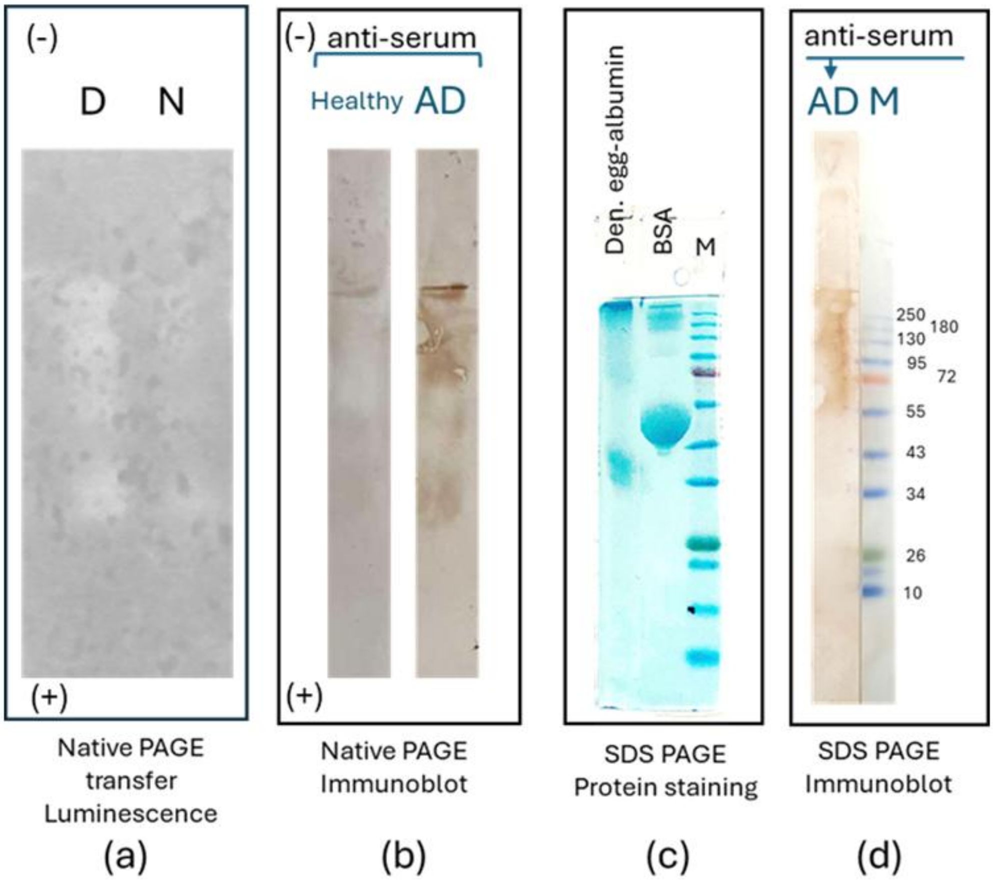

Figure S1. Denatured egg-albumin transfer and immunoblot using anti-serum of healthy and anti-denatured egg-albumin positive AD patients, after native and SDS (Sodium Dodecyl Sulfate) denatured electrophoresis. High molecular weight egg-albumin bands, probable polymerization products, are detected and immunoblot-stained. **(a)** Identification of proteins transferred to nitrocellulose after native electrophoresis of native (N) and denatured (D) egg-albumin. Visualization of fluorescent bands by exposure to UV light of 320nm, a property based on the presence of Trp and Tyr. **(b)** Immunoblot following alkaline native electrophoresis of denatured egg-albumin, using serum of egg-albumin antibody negative healthy individual and dual anti-denatured/anti-native egg-albumin positive anti-serum of Alzheimer's Disease patient (AD), **(c)** SDS electrophoresis of denatured egg-albumin and BSA, stained with amido black, **(d)** Immunoblot following SDS electrophoresis of denatured egg-albumin, using anti- egg-albumin positive anti-serum of Alzheimer's Disease patient (AD). M: Molecular Weight markers. For native electrophoresis, 5 μl of samples were mixed with 5 μl of 2x loading buffer containing 62.5 mM Tris-HCl pH 8.8, Glycerol 25%(v/v) and 0.01% Bromophenol blue and were electrophorized in 10% polyacrylamide gel in Tris-HCl pH 8.8 towards the anode (+). The running buffer consisted of 25mM Tris base and 192mM Glycine (pH 8.8). For SDS-electrophoresis, 5 μl of samples were mixed with 5 μl of 2x loading buffer containing 62.5 mM Tris-HCl pH 6.8, Glycerol 25%(v/v), 0.01% Bromophenol blue and 2% (w/v) SDS and were electrophorized in 10% polyacrylamide gel Tris-HCl buffer pH 8.8. The running buffer consisted of 25mM Tris base, 192mM Glycine and 0.1% SDS (pH 8.3). For native electrophoresis, to diminish denaturation, western blot electro-transfer was carried out in transfer buffer 25 mM Tris, 192 mM glycine, pH 8.3 containing 5% methanol, without SDS. For SDS-electrophoresis Western blot, electro-transfer was performed in transfer buffer containing 0.05% SDS and 20% methanol.
