# Supplementary material for: Increased Concentration of Anti-Egg Albumin Antibodies in Cerebrospinal Fluid and Serum of Patients with Alzheimer’s Disease—Discussion on Human Serpins’ Similarity and Probable Involvement in the Disease Mechanism
Source: Biomolecules. 2025 Jul 27;15(8):1085. doi: 10.3390/biom15081085 (PMC12383860; doi:10.3390/biom15081085)
Supplement: Supplementary file 1 [file biomolecules-15-01085-s001.zip › Supplementary Table S1 last.pdf]

Supplementary Table S1. Characteristics of the AD patients providing the CSF samples for the results presented at Figure 2

Table S1. Characteristics of the AD patients providing the CSF samples, the results of which are presented at Figure 2.

	Age (years)			Education (years)			MMSE		
	**range**	**mean**	**median**	**range**	**mean**	**median**	**range**	**mean**	**median**
**mild**									
all	52-78	76	74	2-19	8.6	6	19-25	22.38	23
female	52-87	78	77	2-17	7.7	6	19-25	22.26	21
male	59-85	74	73	6-19	9.5	7	20-24	22.50	23
**severe**									
all	57-89	76	78	2-16	7.4	7	0-10	5.35	5
female	57-89	74	78	2-14	6.9	7	0-10	5.80	5
male	64-86	79	78	3-16	8.1	8	0-10	5.00	5
