# Supplementary material for: Increased Concentration of Anti-Egg Albumin Antibodies in Cerebrospinal Fluid and Serum of Patients with Alzheimer’s Disease—Discussion on Human Serpins’ Similarity and Probable Involvement in the Disease Mechanism
Source: Biomolecules. 2025 Jul 27;15(8):1085. doi: 10.3390/biom15081085 (PMC12383860; doi:10.3390/biom15081085)
Supplement: Supplementary file 1 [file biomolecules-15-01085-s001.zip › Supplementary Table S2 last.pdf]

Supplementary Table S2. Characteristics of the AD patients providing serum samples for the results presented at Figure 3.

Table S2. Characteristics of the AD patients providing serum samples, the results of which are presented at Figure 3

	Age (years)			Education (years)			MMSE		
	**range**	**mean**	**median**	**range**	**mean**	**median**	**range**	**mean**	**median**
**mild**									
all	65-89	78	78	2-16	9,15	9	20-26	22,6	22
female	65-89	78	76	2-14	8,45	8	20-25	22,55	22
male	72-86	79	79	6-16	10	9	21-26	22,67	22
**intermediate**									
all	55-87	76	78	2-19	8,4	6	14-20	17,35	18
female	73-87	78	77	2-12	7,3	6	14-20	17	18
male	55-83	75	79	3-19	9,2	7	14-20	17,6	18
**severe**									
all	61-90	77	78	1-13	7,1	6	2-13	7,95	7
female	61-90	77	78	1-13	6,3	6	2-13	8	8
male	62-88	75	77	6-12	8,6	9	3-11	7,85	7
