# Supplementary material for: Increased Concentration of Anti-Egg Albumin Antibodies in Cerebrospinal Fluid and Serum of Patients with Alzheimer’s Disease—Discussion on Human Serpins’ Similarity and Probable Involvement in the Disease Mechanism
Source: Biomolecules. 2025 Jul 27;15(8):1085. doi: 10.3390/biom15081085 (PMC12383860; doi:10.3390/biom15081085)
Supplement: Supplementary file 1 [file biomolecules-15-01085-s001.zip › Supplementary text S1 last.pdf]

Supplementary text S1: "Involvement of serpins with high sequence identity with egg-albumin in AD pathophysiology"

Intestinal inflammation and permeability

The serpin **B1** sharing 38.8% identity with ovalbumin, is a serpin secreted by the neutrophils [74], responsible for cell protection during inflammation with an involvement in maintenance of endothelial cell junction integrity. Although it belongs to the family of serin protease inhibitor, it can inhibit both serine and cysteine proteases among which elastase, cathepsin G, proteinase 3 and chymase that are secreted by immune cells such as neutrophils and mast cells during inflammation, a common disorder in A.D. [75, 76]. Among the above proteases, chymase can enhance epithelial permeability by causing redistribution of the tight junction proteins ZO-1 and occludin [77, 78]. Serpines **A2** and **A3** with 28.1% and 30.0% identity, respectively, also act as cathepsin and chymase inhibitors. The intestinal equilibrium between proteases and serpins is of great importance for intestinal health and proper function. As suicide inhibitors, serpins strongly bind to their target proteases forming covalent complexes (SEC) [79]. Elimination of the serpin-protease complexes and regulation of secretion of serpins is done through interaction of the Serpin-Enzyme Complex (SEC) with specific SEC-receptors and endocytosis [80]. The common form of ovalbumin cannot substitute intestinal serpins in their interactions, although it belongs to the serpin family. Only the rare I-form of heat-denatured egg-albumin which acquires the inhibitory property under specific denaturing conditions could act as reversible inhibitor, mimicking serpins' activity and their ability to interact with SEC receptors. However, this is a rare form of albumin and there is no evidence that such a form exists in the intestine [79]. Antibodies recognizing the serpins' binding site to SEC receptors may prevent the serpin interaction, impairing control of serpin secretion and activity [80]. Cross-reacting anti-ovalbumin antibodies could theoretically implicate in this process, contributing to intestinal inflammation and impaired permeability.

Blood Brain Barrier

The serpin **A8**, known as angiotensin (AGT) is present both in the circulation and in the brain, with lower levels of circulating AGT present in AD patients compared to healthy population [81]. Apart from the main role of AGT and its active metabolite, angiotensin II (AngII), in regulating blood volume and arterial pressure, a number of CNS functions have also been known, including control of cerebral blood flow and involvement in memory retention and neuronal regeneration. In the area of Blood Brain Barrier, AngII of brain but also of plasma origin [82] interacts with the angiotensin receptor 1 (AGTR1) to restrict the passage through BBB epithelial cells by controlling the action of occluding [70]. In the brain, AGT is produced locally by astrocytes mainly acting via AGTR2 and may have a role in cognition and brain health [71,83].

CNS

Proteolytic mechanism is crucial in AD development. Several proteases among which a-secretase, β-secretase and γ-secretase as well as proteases involved in their maturation and clearance are closely connected to beta-amyloid production and degradation. Consequently, the balance of protease inhibitors is of great importance. It is well known that the normal Aβ peptide is produced when the intramembrane amyloid-beta precursor protein (AβPP) is sequentially hydrolyzed by the extracellular a- and γ-secretases while the action of β-secretase results to the production of the shorter abnormal Aβ peptide which then aggregates to give

the amyloid plaques [24]. Thus, decrease of α-secretase and increase of β-secretase activity may be among the events leading to AD dysregulation, affecting the levels of normal and pathologic Aβ peptides. Moreover, imbalance in the activity of proteolytic enzymes involved in maturation of α- or β- secretases may be related to the disease and so is with the enzymes implicated in the Aβ peptide clearing. Therefore, proteases' inhibitors may have a central role in AD development and progression and antibodies directed against such inhibitors may alter substantially the normal process. Serine protease inhibitors (serpins) constitute a great category of proteases. The main AD related proteases α-, β- and γ-secretases could not be directly affected by serpines, since they are aspartate and not serine proteases. However, β-secretase activity could be indirectly affected, through a probable increase in the action of fucin which possesses serine protease activity and is believed to participate in β-secretase maturation [84]. On the other hand, several serine proteases among which plasmin and akylpeptide hydrolase, with different impact in natural Aβ and Aβ-fibril degradation, are among the Aβ peptide clearing enzymes, although they constitute a minority of the proteolytic enzymes involved [24,85].

Recent studies have shown upregulation of serpin **A1** (a1-antitrypsin), **A3** (a1-antichymotrypsin) and **B1** in the brain of patients with Alzheimer's Disease. Among these, the expression of **serpin A3** is much higher than that of the others [69,86]. It is believed that upregulation may be a response to the elevated protease activity, which increases to counteract the accumulation of disease–related protein aggregates [87]. However, it may also be increased as a response to apoE disfunction. Although, increased apoE is observed in all AD patients, the increase is much higher in patients with the AD-related apoE4 variant. As concluded by animal studies, lack of normal apoE interaction with its receptors is correlated with increased **serpin A3**. The increased expression of serpin A3 in apoE-knock-out mice is normalized by insertion of the apoE3 human allele but not by insertion of the apoE4 allele [88]. The main difference between the two alleles is the increased susceptibility of the apoE4 allele to hydrolysis by chymotrypsin-like proteases [89]. According to these findings, the increase in **serpin A3** could be a natural reaction to counteract a dysregulation related to apoE and processes which could lower the serpinA3 concentration would augment the imbalance. It is of interest to explore if antibody cross-reaction could be among these processes. Interestingly, immunohistochemical analysis also revealed the presence of **serpinA3** in activated astrocytes during aging, in contrast to the minimal expression in healthy, young individuals [90].

Serpin **I1** (neuroserpin) is a secretory protein [91], involved in the formation or reorganization of synaptic connections. It is related to synaptic plasticity in the adult nervous system and may protect neurons from cell damage by tissue-type plasminogen activator. Mutations leading to decreased inhibitory activity or reduced stability of neuroserpin are the cause of familial dementia [92].

Serpin I1, has been related to AD, seeming to have a pivotal role. It binds to the amyloid Aβ peptide with which it can form 1:1 complex, leading to non-fibrillary, less toxic Aβ species [93]. Under this concept, neuroserpin is considered to have a neuroprotective effect. Decreased mRNA and protein neuroserpin levels were observed in post-mortem studies of severe AD-patients (Braak stage IV-VI) [94]. However, increased [96] serpin concentration in the AD brain has also been mentioned, attributing to neuroserpin a negative role through the effect of increased concentration on the plasminogen activator (tPA)-plasmin axis and on the Aβ peptide clearance. Reduced tPA and plasmin which is the main protease involved in Aβ clearance, is observed in AD brain [95]. However, neuroserpin is not the only tPA inhibitor as

it shares this property with Plasminogen Activator Inhibitor (PA-I1) [96]. PAI-1 is also a serpin (serpin **E1**). Interestingly, references mentioning increased concentrations of neuroserpin in AD brain, mention stable concentrations of PAI-1 [97], while references mentioning decreased concentrations of neuroserpin, mention PAI-1 elevation [94], but until now there is no evidence for the etiology beneath this controversy. Increased neuroserpin may be the result of uncontrolled neuronal excitation [98]. Moreover, the activation of adaptive immune response in AD patients, characterized by proportional increase of all immune cells with disease severity [99], may explain increases in neuroserpin concentrations, since it is secreted by the immune cells [94].

A number of other serpins with similarity to ovalbumin are known to act in the brain affecting neuron function. Among them, SERPIN **F1** is related to short-term memory, positive regulation of neurogenesis [100], neuron projection development, negative regulation of neuron death and inflammation. Moreover, recent studies have connected AGT [101] with long-term neuronal synaptic plasticity and regulation of transmission of nerve impulse while angiotensin-(1-7) peptide is believed to be involved in associative learning.

References

In the main article "Dionysia Amanatidou, Magdalini Tsolaki, Vasileios Fouskas, Ioannis Gavriilidis, Maria Myriouni, Anna Anastasiou, Athanasia Papageorgiou, Diona Porfyriadou, Zoi Parcharidi, Eleftheria Papasavva, Maria Fili, Phaedra Eleftheriou*. **Increased concentration of anti-egg albumin antibodies in cerebrospinal fluid and serum of patients with Alzheimer's Disease - Discussion on human serpins' similarity and probable involvement in the disease mechanism**, *Biomolecules*, **2025**, *15*, ."
